# 

**Published:** 1842-10

**Authors:** 


### WESTERN JOURNAL

OF

### MEDICINE AND SURGERY.

### EDITORS.

---

DANIEL DRAKE, M. D.,

PROFESSOR OF PATHOLOGICAL ANATOMY AND CLINICAL MEDICINE

IN THE MEDICAL INSTITUTE OF LOUISVILLE:

LUNSFORD P. YANDELL, M. D.,

PROFESSOR OF CHEMISTRY AND PHARMACY IN THE SAME:

THOMAS W. COLESCOTT, M. D.

### DELINQUENT LIST.

It will be seen that we continue our list of delinquents, which a press of matter last month prevented. Next month the list will be completed, and it will behoove all in arrearages, who are not desirous of having their names appear *in print*, to remit the amount of their accounts without delay. Post Masters will frank remittances.

TENNESSEE.

J. Drake, South Carroll
J. J. Gibson, Hampshire
Wilson & Bearden, Petersburgh
L. M. N. Cook, Silver Spring
T. J. Walton, Mulloy's
S. B. Robinson, Versailles
J. M. Dean, Lynchburg
A. F. Clement, Lexington
W. H. Warren, Lexington
W. T. Eatherty, Rural Hill
J. A. Lecky, Ripley
S. Richardson, Rutherford.
W. D. Lee, Rutherford.
W. J. J. Morrow, Chattawogga
J. W. Koen, Colliersville
J. M. M. Cornelius, Germantown
Robinson & Hagan, Carthage
J. B. Copeland, Nolandsville
R. Forbis, Elkton
M. W. O'Brien, Springfield.
W. J. C. Rogers, Murfreesboro'

NEW YORK.

Wm. Welch, Penfield
G. Standing, Utica

ARKANSAS.

W. A. Threlkeld, Helena
J. H. McCarty, Ozark
Dr. Beach, Lawrenceville
J. Eaton, Pine Bluff

LOUISIANA.

B. B. Smith, Shreveport
J. B. Henderson, New Orleans.
J. Davis, Millekin's Bend
T. Ingersoll, Cherryville
E. Ragan, Columbia

ALABAMA.

P. L. Gilbert, Isny, Washington County.
R. L. Graves, Leesville
D. W. Ford, Meridianville
E. M. Hussey, Waterloo
W. R. Davie, Waterloo
W. F. Johnson, Benton
J. Durno, Mobile, or Alton, Ill.
J. T. Reese, Syllacogga.

MISSISSIPPI.

Harris & McLaen, Wahallock
E. F. Bouchelle, Columbus
S. D. Kennedy, Holmesville
D. B. Nailer, Vicksburgh
—— Hogan, Panola
A. P. Reed, Fort Adams
J. A. Hodges, Garlandsville
O. S. Echols, Hamilton
L. Shelby, Warrenton

ILLINOIS.

O. Abbey, Quincy
W. H. Efner, Lacon
J. W. Chenoweth, Auburn
C. L. Duncan, Marshall
J. R. Colwell, Farmington
J. Kirkpatrick, Fox P. O.
W. Allison, Paradise
W. Patton, Oakland
T. J. Beckwell, Rushville
J. Jayne, Carlinsville
B. F. Edwards, Alton
C. F. Falley, York
H. Symmes, Equality
W. W. Higgins, Jones' P. O.
W. O. Chamberlain, Princeton
Wood & Goodwin, Holland's Grove
F. Canterbury, Cole's C. H.
H. P. Griswold, Plymouth
J. M. Metcalf, Winchester
W. Kellar, Decatur

INDIANA.

B. F. Russell, New Washington
J. H. McNutt, Annapolis
D. Long, Blackford
D. Hoover, Wolf Lake
J. L. Plaff, Westfield
F. C. Gale, Vevay
N. B. Odelle, Lafayette
J. M. Bell, Dublin
P. Q. Stryker, Rockville.
W. Anderson, Somerset
W. H. Martin, Rushville
C. West, Richmond
L. Edwards, Greenfield
J. A. Pomeroy, Roseville
E. H. Bruce, Peru
S. P. Wort, Brownstown.
J. Prosser, Orleans
H. Single, Orleans
T. Losey, Laporte
W. Simpkins, Lebanon
W. M. Gentry, Frankfort
W. V. Snyder, Frankfort
R. Hobson, New Harmony
J. H. Herryman, Bowling Green
D. Todd, Monticello
A. Poteet, Monticello
C. Wallace, Indianapolis
S. T. Clark, Russelville
J. W. Porter, Greenville
J. C. Clarke, Greenville
T. Sturges, Fort Wayne
A. D. Sweet, Franklin
E. S. Blue, Leesburgh
J. Darr, New Castle
J. V. Wayman, New Castle
J. W. Crooks, Rockport
J. W. French, Troy
A. M. Murphy, Bloomington
I. N. Griffith, Jeffersonville
D. C. McGee, Mount Pleasant
J. McCallen, McAllen's Cross Roads
J. S. Deputy, Madison
—— Tilton, Madison
S. Wyatt, Lexington
W. W. Goodwin, Charlestown
G. R. McCoy, Charlestown

---

The Western Journal of Medicine and Surgery is published monthly by the undersigned, at the corner of Main and Fifth streets, Louisville, at $5 per annum, payable in advance. Each number contains from 80 to 84 pages making two volumes in the year of about 500 pages.

Contributions to its pages by the Physicians of the Valley of the Mississippi, addressed to the Editors, are respectfully solicited.

Letters on business, to be addressed, postage paid, to the publishers.

☞Postmasters, by the regulations of the Postoffice Department, will frank letters containing subscription money, and all remittances so franked are at the risk of the publishers.

*August*, 1842. PRENTICE & WEISSINGER.

